# Utility of beta‐human chorionic gonadotropin in pleural effusions: Report of an autopsy case of a male patient with primary pulmonary choriocarcinoma

**DOI:** 10.1002/ccr3.6663

**Published:** 2022-12-05

**Authors:** Kazuhiko Iwasaki, Kazuyoshi Watanabe, Hideharu Kimura, Seiji Yano

**Affiliations:** ^1^ Department of Respiratory medicine Japan Community Health Care Organization Kanazawa Hospital Kanazawa Japan; ^2^ Department of Respiratory medicine Kanazawa Graduate school of Medical Sciences Kanazawa Japan

**Keywords:** bloody pleural fluid effusion, choriocarcinoma, β‐Human chorionic gonadotropin

## Abstract

A 69‐year‐old male patient presented with bloody pleural fluid effusion and elevated beta‐human chorionic gonadotropin (β‐hCG) levels obtained by thoracentesis. The patient's condition rapidly deteriorated, and he died. The autopsy revealed primary pulmonary choriocarcinoma. Early diagnosis of choriocarcinoma based on β‐hCG levels in the pleural fluid may be possible.

## Graphical abstract

We report the case of a male patient with primary pulmonary choriocarcinoma who died within a short period. Bloody pleural fluid effusion with high human chorionic gonadotropin levels may be used as a marker for early diagnosis of choriocarcinoma.

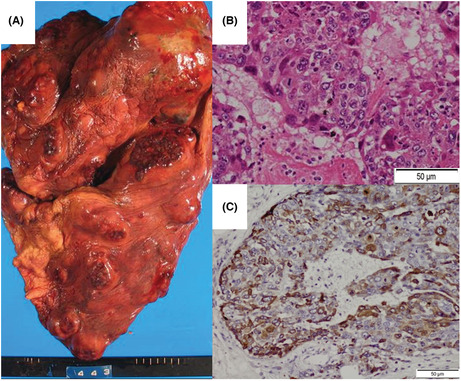

## 1 INTRODUCTION

Choriocarcinoma usually occurs in the genital organs but rarely in the mediastinum, lung, stomach, and pineal gland. Primary pulmonary choriocarcinomas (PPC) are rare, with only 65 cases reported to date.^1^ The preoperative diagnosis of PPC in males is difficult, and most cases are diagnosed using surgical or autopsy specimens.

Here, we describe the rapidly progressing clinical course of a male patient with primary pulmonary choriocarcinoma and suggest that β‐human chorionic gonadotropin (β‐HCG) measurement in pleural fluid effusion may contribute to early diagnosis.

## 2 CASE PRESENTATION

**FIGURE 1:**
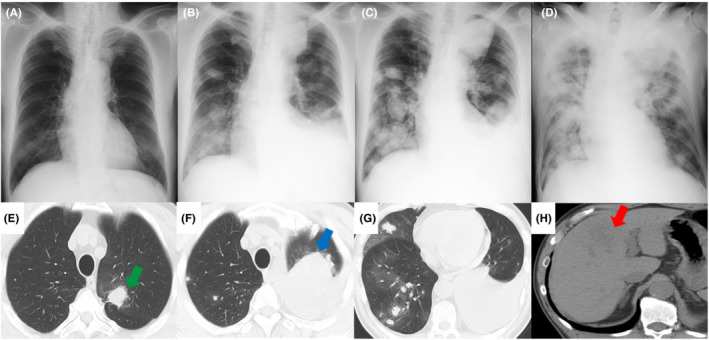
(A) Chest radiograph (CR) obtained 3 months prior to admission. (B) CR on admission showing a left upper lobe mass shadow, left pleural effusion, and multiple intrapulmonary metastases. (C) CR on Day 10 of hospitalization, showing an overall worsening of shadows. (D) CR on Day 14 of hospitalization, showing the patient in a supine position due to dyspnea, with worsening shadows. (E) Computed tomography (CT) scan obtained 3 months prior to admission. (F–H) CT scan on admission.

**FIGURE 2:**
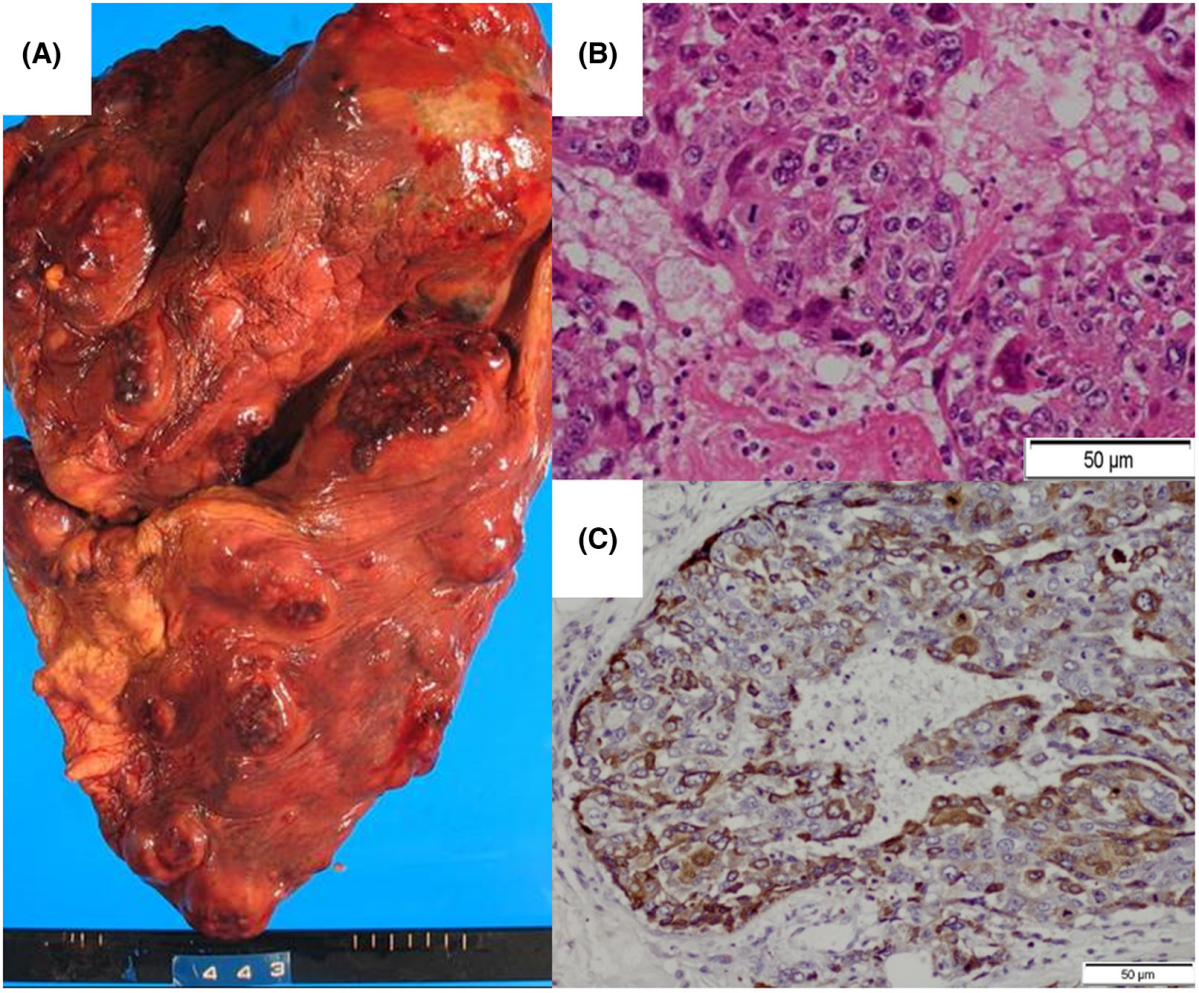
(A) The left lung showing protruding masses on both sides of all lobes when it was removed for autopsy. (B) Histology showing Langhans‐like atypical cells surrounding syncytial cell‐like atypical cells, resembling immature villi. (C) Immunostaining is positive for hCG. Immunostaining for NapsinA, ChromA, TF‐1, Synapto, CK5/6, p40, CK20, and CD56 were all negative (data not shown).

A 69‐year‐old man with bronchiectasis, hypertension, hyperlipidemia, and a history of smoking 13 packs per year visited another hospital 3 months earlier for left shoulder pain and was found to have a 20 mm large left upper lung lobe nodule (Figure 1A,E; green arrow). A further detailed examination was scheduled there, but was postponed for his own reasons. At this time, he visited our hospital because of mMRC3 dyspnea and chest pain. Since there had been no falls or other injuries in the previous 6 months, trauma was ruled out. Chest radiography and computed tomography (CT) scan on admission revealed a 60 mm large mass shadow (Figure 1B,F; blue arrow), indicative of a primary tumor and multiple metastatic tumors in the bilateral lungs (Figure 1G). In addition, a liver metastatic tumor was observed (Figure 1H; red arrow). On admission, pleural effusion was performed, and a bloody effusion was obtained, but only a few atypical cells were found in the effusion. The same pathological findings were observed from a total of three different examinations performed at different sites. Bronchoscopy revealed no abnormalities in the bronchial lumen, and biopsy of the left upper lobe showed no atypical cells. The patient showed rapid pleural effusion within 2 weeks of admission (Figure 1C,D), and his dyspnea, oxygenation, pain, worsened, and his performance status deteriorated markedly. Therefore, morphine was chosen by his family to relieve pain and dyspnea. On Day 18, the patient died of respiratory failure. The autopsy revealed multiple masses in the visceral pleura of both lungs, predominantly in the left lung where there were also some areas of organic degeneration. No testicular tumor was found, and after pathological examination, a diagnosis of PPC was made with no evidence of double carcinoma (Figure 2B,C). An abnormally high level of β‐hCG was detected in the pleural fluid, and serum samples collected during hospitalization (Table 1).

**TABLE 1:** Laboratory findings

Blood		Pleural effusion examination	
Estradiol	56.7 pg/ml	Estradiol	92.3 pg/ml
Progesterone	1.5 ng/ml	Progesterone	2.6 ng/ml
Testosterone	1.91 ng/ml	Testosterone	1.08 ng/ml
Human placental lactogen	<0.07 μg/ml	Human placental lactogen	0.259 μg/ml
Alpha fetoprotein	<2.0 ng/ml	Alpha fetoprotein	<2.0 ng/ml
Beta‐human chorionic gonadotropin	27,000 mIU/ml	Beta‐human chorionic gonadotropin	3624 mIU/ml
Carcinoembryonic antigen	3.8 ng/ml	Glucose	123 mg/dl
Cytokeratin 19fragment	95.6 ng/ml	pH	7.3
Pro‐gastrin releasing peptide	49.2 pg/ml	Adenosine deaminase	8.4 U/I
Blood sugar	114 mg/dl	Bacterial culture test	Negative

## 3 DISCUSSION AND CONCLUSION

Choriocarcinoma is a disease characterized histologically by sheets of cellular trophoblastic and syncytial trophoblastic cells^2^ and is diagnosed by high serum β‐hCG levels and positive immunostaining.^3^

The prognosis of this tumor is extremely poor despite surgical treatment and chemotherapy.^4^

However, since there are many reports that surgery and postoperative chemotherapy are effective,^5^, ^6^, ^7^ early diagnosis is important.

This case illustrates two important clinical points.

First, choriocarcinoma is considered a tumor tissue with high blood flow^8^ and can thus cause bloody effusions. Therefore, it should be considered as a differential diagnosis when the patient has bloody pleural effusion.

Second, in choriocarcinoma, tumor samples are small and cytology is difficult because of hemorrhage and necrosis.^9^ A high β‐hCG level in a pleural fluid sample may be useful for early diagnosis, as is a high β‐hCG level in a serum sample.

It should be noted that large cell carcinoma,^10^ pleomorphic carcinoma,^11^ and adenocarcinoma of the lung^12^ have been reported as β‐hCG‐producing lung tumors; however, without histological examination, it is difficult to differentiate them. There have also been reports of choriocarcinoma and lung cancer complications, making it even more difficult to distinguish the two in cases of rapid progression.^13^ There is agreement that early surgery or chemotherapy improves the prognosis of these diseases, especially in the case of PPC.

Therefore, in cases where a bloody pleural effusion is obtained, the measurement of β‐hCG is recommended, suggesting that it may aid in early diagnosis and treatment.

## AUTHOR CONTRIBUTIONS

KI wrote the initial draft of the manuscript and was responsible for drafting and image modification. KI, KW, HK, and SY were directly involved in treatment, critically revised the manuscript, and approved the final version.

## FUNDING INFORMATION

This research was not supported by any specific grant from any funding agency in the public, commercial, or nonprofit sectors. Therefore, no funding body was involved in the study design, collection, analysis, and interpretation of the data, writing of the manuscript, or decision to submit the manuscript for publication.

## CONFLICT OF INTEREST

None.

## CONSENT

Consent for publication was obtained from living patient prior to submission.

## Data Availability

No datasets were generated or analyzed for this case report.
